# DPYSL2 as potential diagnostic and prognostic biomarker linked to immune infiltration in lung adenocarcinoma

**DOI:** 10.1186/s12957-021-02379-z

**Published:** 2021-09-13

**Authors:** Yang-Jie Wu, Ai-Tao Nai, Gui-Cheng He, Fei Xiao, Zhi-Min Li, San-Yuan Tang, Yan-Ping Liu, Xiao-Hong Ai

**Affiliations:** 1 Department of Radiation Oncology, The First Affiliated Hospital of University of South China, Hengyang, 421001 China; 2 Department of Oncology, The First Affiliated Hospital of Jinan University, Guangzhou, 510632 China; 3 Department of Oncology, The First Affiliated Hospital of University of South China, Hengyang, 421001 China; 4 Department of Oncology, Brain Hospital of Hunan Province, Changsha, 410007 China

**Keywords:** DPYSL2, Prognosis, Lung adenocarcinoma, Immune infiltration, Bioinformatic analysis

## Abstract

**Background:**

Dihydropyrimidinase like 2 (DPYSL2) has been linked to tumor metastasis. However, the function of DPSY2L in lung adenocarcinoma (LUAD) is yet to be explored.

**Methods:**

Herein, we assessed DPYSL2 expression in various tumor types via online databases such as Oncomine and Tumor Immune Estimation Resource (TIMER). Further, we verified the low protein and mRNA expressions of DPYSL2 in LUAD via the ULCAN, The TCGA and GEPIA databases. We applied the ROC curve to examine the role of DPYSL2 in diagnosis. The prognostic significance of DPYSL2 was established through the Kaplan–Meier plotter and the Cox analyses (univariate and multivariate). TIMER was used to explore DPYSL2 expression and its connection to immune infiltrated cells. Through Gene Set Enrichment Analysis, the possible mechanism of DPYSL2 in LUAD was investigated.

**Results:**

In this study, database analysis revealed lower DPYSL2 expression in LUAD than in normal tissues. The ROC curve suggested that expression of DPYSL2 had high diagnostic efficiency in LUAD. The DPYSL2 expression had an association with the survival time of LUAD patients in the Kaplan–Meier plotter and the Cox analyses. The results from TIMER depicted a markedly positive correlation of DPYSL2 expression with immune cells infiltrated in LUAD, such as macrophages, dendritic cells, CD4+ T cells, and neutrophils. Additionally, many gene markers for the immune system had similar positive correlations in the TIMER analysis. In Gene Set Enrichment Analysis, six immune-related signaling pathways were associated with DPYSL2.

**Conclusions:**

In summary, DPYSL2 is a novel biomarker with diagnostic and prognostic potential for LUAD as well as an immunotherapy target.

**Highlights:**

1. Expression of DPYSL2 was considerably lower in LUAD than in normal tissues.
2. Investigation of multiple databases showed a high diagnostic value of DPYSL2 in LUAD.
3. DPYSL2 can independently predict the LUAD outcomes.
4. Immune-related mechanisms may be potential ways for DPYSL2 to play a role in LUAD.

## Background

Globally, lung cancer accounts for the highest number of tumor-related deaths [1]. The most prevalent pathologic type of non-small cell lung cancer, lung adenocarcinoma (LUAD), constitutes nearly 85% of entire cases [2, 3]. Despite the availability of multiple therapies, the rate at which LUAD patients can survive for 5 years is 15% [1, 4]. Recent advancements in tumor immunotherapy have prompted the continuous update of treatment models for many types of cancer [5–8]. Immune-related mechanisms have also been instrumental in LUAD [9]. Numerous studies have shown that immunotherapeutic approaches, among them, programmed death-1 inhibitors exhibit high tolerability and anti-tumor effects when treating tumors [8, 9]. However, these immunotherapy drugs have some drawbacks, including high costs and limited benefits for specific cancer patient cohorts [10]. Compelling evidence indicates that immune cells infiltrating tumors are linked to immunotherapy efficacy and that biomarkers found in immune cells have significant implications for patient outcomes [11–14]. Therefore, further research is needed to explore new immune-related biomarkers.

Collapsin response mediators are homo- and hetero-tetrameric proteins that play a role in Sema3A-driven growth cone collapse, cell migration, and promote neuron guidance, development, and polarity [15–18]. Numerous reports have recently implicated DPYSL2 phosphorylation in the development of drug resistance and tumor metastasis, but there are a few reports on the involved mechanisms [19, 20]. For example, a recent study demonstrated that DPYSL2 could inhibit stemness and metastasis of cancer cells in breasts through stabilization of kazal motifs-harboring proteins, e.g., reversion-inducing cysteine-rich proteins [21]. However, there are few reports on DPYSL2 in other tumors.

Herein, through databases such as Oncomine, TIMER, and others, we explored the association of DPYSL2 expression with LUAD prognosis of patients. Moreover, DPYSL2-immune infiltration associations were assessed with TIMER. The GSEA was applied in the TCGA-LUAD dataset, which revealed the potential molecular mechanism for DPYSL2. Our findings demonstrated the prognostic function of DPYSL2 expression in LUAD patients, and the possible correlation and interaction mechanism between DPYSL2 and immune response in tumors.

## Materials and methods

### Data collection

Gene expression profiles and clinical details of 585 LUAD patients were retrieved from the TCGA database using Xena browser (https://xenabrowser.net/datapages/). The TCGA-LUAD cohort contained information on 59 normal tissues and 526 adenocarcinoma tissues (13 of which were duplicated).

### Oncomine database analysis

We used Oncomine, a comprehensive database for the study of tumor-related genes to assess the level of DPYSL2 expression in different cancer types [22]. In Oncomine database, the specified gene was assessed for differential expression with Student’s *t* test. *p* value < 0.05, fold change > 2, gene ranking = all, data type = all were set as the threshold.

### DPYSL2 gene expression analysis

In TCGA-LUAD, the statistical significance of the expression levels of DPYSL2 was tested in 513 LUAD tissues and 59 surrounding normal tissues using unpaired and paired *t* test. *P* < 0.05 was considered statistically meaningful. The GEPIA database was employed for the analysis of the DPYSL2 showing differential expression. GEPIA is a developed interactive website that integrates TGCA data and data from the Genotype-Tissue Expression projects [23].

### Analysis of DPYSL2 protein expression

The immunohistochemical images of DPYSL2 protein in lung adenocarcinoma and healthy lung tissues were derived from the HPA database [24]. Moreover, the difference in protein expression of DPYSL2 between normal tissues and LUAD tissues was analyzed via the UALCAN database, a bioinformatics tool [25, 26].

### Survival analysis of DPYSL2 expression

We employed the Kaplan–Meier plotter database for analysis of DPYSL2’s prognostic role in LUAD patients [27]. Using the progression-free survival (PFS, *n* = 461) and the overall survival (OS, *n* = 719) of LUAD patients, we established the prognostic value of DPYSL2 expression. Accordingly, the median value expressed by DPYSL2 guided us in categorizing LUAD patients into high and low groups. The 95% confidence intervals (CI), hazard ratio (HR), and *log-rank P* values were then determined. In addition, “survminer” and “survival” packages in R (version 4.0.3) were applied to examine the connection between DPYSL2 level and OS in TCGA-LUAD data.

### TIMER database analysis

The TIMER is an online resource that integrates 10,897 samples across 32 types of cancer in the TCGA, allowing researchers to systematically evaluate how different immune cells clinically impact various cancers [28, 29]. We applied the TIMER to explore DPYSL2 expression and its connection to several immune system cells (dendritic cells, macrophages, CD8+ T cells, B cells, CD4+ T cells, and neutrophils) in LUAD. The purity of the tumor was also examined because it is crucial for determining immune infiltration [30]. Furthermore, we applied the correlation modules of TIMER to evaluate DPYSL2 level association with genes of immune infiltrated cells.

### Gene set enrichment analysis

Using the GSEA, we explored the possible mechanism of DPYSL2 by analyzing the TCGA-LUAD dataset with c5.all.v7.2 ontology gene sets from Molecular Signatures Database [31]. The median values expressed by DPYSL2 were applied in categorizing the TCGA-LUAD cohort into two groups (high and low). *P* adjust value < 0.05 denoted statistical significance.

### Statistical analysis

All statistical data were analyzed in R software (version 4.0.3). We applied paired *t* test and unpaired *t* test for gene expression differential analysis. The diagnostic value of DPYSL2 in LUAD was assessed via the receiver operating characteristic (ROC) curves. The prognostic value of DPYSL2 in LUAD was delineated via the Kaplan–Meier plotter database. In addition, the prognostic function of DPYSL2 expression was evaluated through multivariate and univariate Cox analyses. *P* value < 0.05 represented statistically significant data.

## Results

### Low DPYSL2 expression in LUAD

**Fig. 1:**
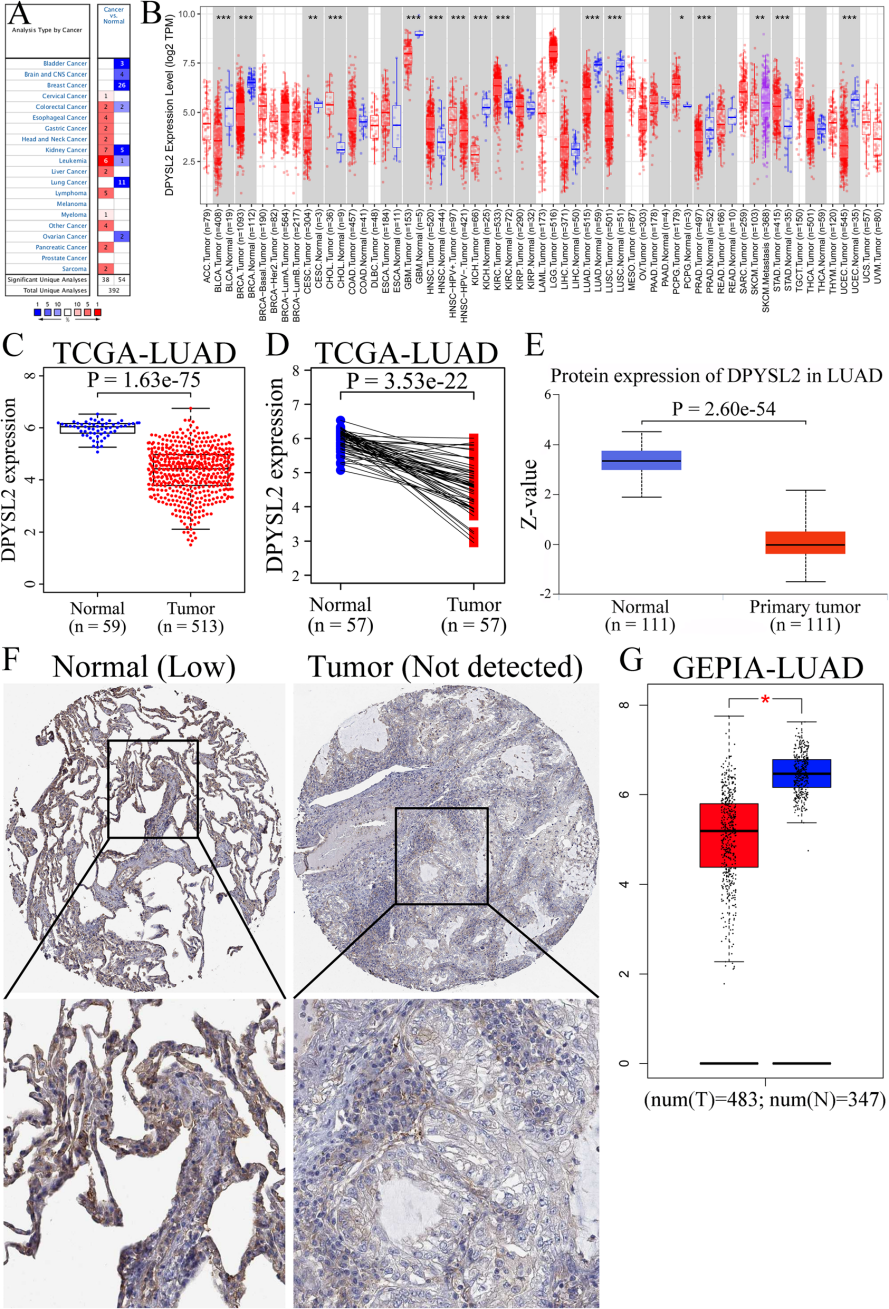
Expression levels of DPYSL2 in LUAD according to various databases. The expression of DPYSL2 in various cancers in Oncomine database (**A**), DPYSL2 expression in various tumors established via TIMER (**B**), DPYSL2 expression in LUAD vs normal lung tissues in the TCGA-LUAD cohort (**C**), expression levels of DPYSL2 in paired LUAD samples in the TCGA-LUAD cohort (**D**), DPYSL2 protein expression in LUAD vs normal lung tissues in the CPTAC-LUAD cohort (**E**), DPYSL2 protein expression in LUAD and adjacent normal lung tissues in the HPA database (**F**), DPYSL2 expression in LUAD vs normal lung tissues in the GEPIA-LUAD cohort (**G**)

The exploration of the Oncomine database revealed markedly lower DPYSL2 level in lung cancer tissues than normal tissues (Fig. 1A). Furthermore, analysis of the TIMER online database indicated a significant downregulation of DPYSL2 expression in LUAD than in the paracancerous tissues or normal lung tissues (Fig. 1B; *P < 0.05, **P < 0.01, ***P < 0.001).

Furthermore, we analyzed DPYSL2 expression in LUAD, whereby the TCGA-LUAD data was retrieved and applied to assess variations in mRNA levels. The unpaired *t* test (Fig. 1C; *t* value = 29.09, *P* = 1.63e−05) and paired *t* test (Fig. 1D; *t* value = 13.60, *P* = 3.53e−22) demonstrated low DPYSL2 expression in LUAD. Analyzing the GEPIA-LUAD cohort yielded similar results (Fig. 1G; *P* < 0.05). Subsequently, we determined the protein level of DPYSL2 from HPA and CPTAC data by *t* test. As expected, we found lower DPYSL2 protein expression in LUAD than in normal tissues (Fig. 1E, F; *P* = 2.60e−54).

### DPYSL2 has a high diagnostic efficiency

**Fig. 2:**
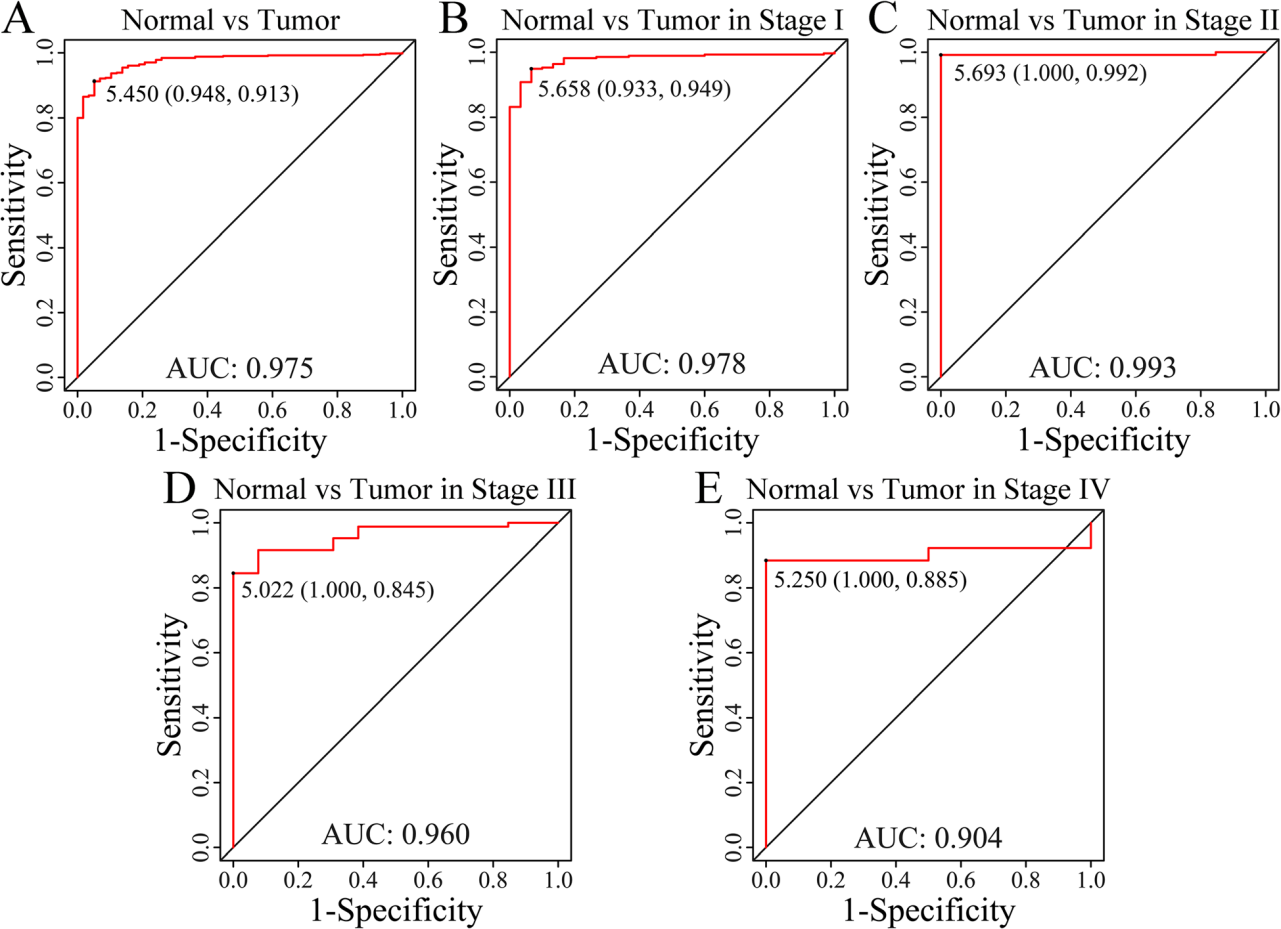
Diagnostic value of DPYSL2 in LUAD. Receiver operating characteristic curve of DPYSL2 expression in LUAD vs normal tissues (**A**) and different clinical stages (**B**–**E**)

We generated ROC curves for the assessment of the diagnostic value of DPYSL2. The entire AUC for DPYSL2 was 0.975 (95% CI 0.962–0.988), suggesting that DPYSL2 was capable of discriminating between adjacent tissues and LUAD tissues (Fig. 2A). Additionally, subgroup analysis showed that the diagnostic value of DPYSL2 in I–IV stages of LUAD had AUC values of 0.978 (95% CI 0.962–0.9995), 0.993 (95% CI 0.979–1.000), 0.960 (95% CI 0.922–0.997), and 0.904 (95% CI 0.789–1.000) respectively (Fig. 2B–E). These findings suggest that DPYSL2 exhibits high diagnostic efficiency in separating patients with LUAD from healthy subjects.

### Low DPYSL2 level predict poor prognosis in LUAD

**Fig. 3:**
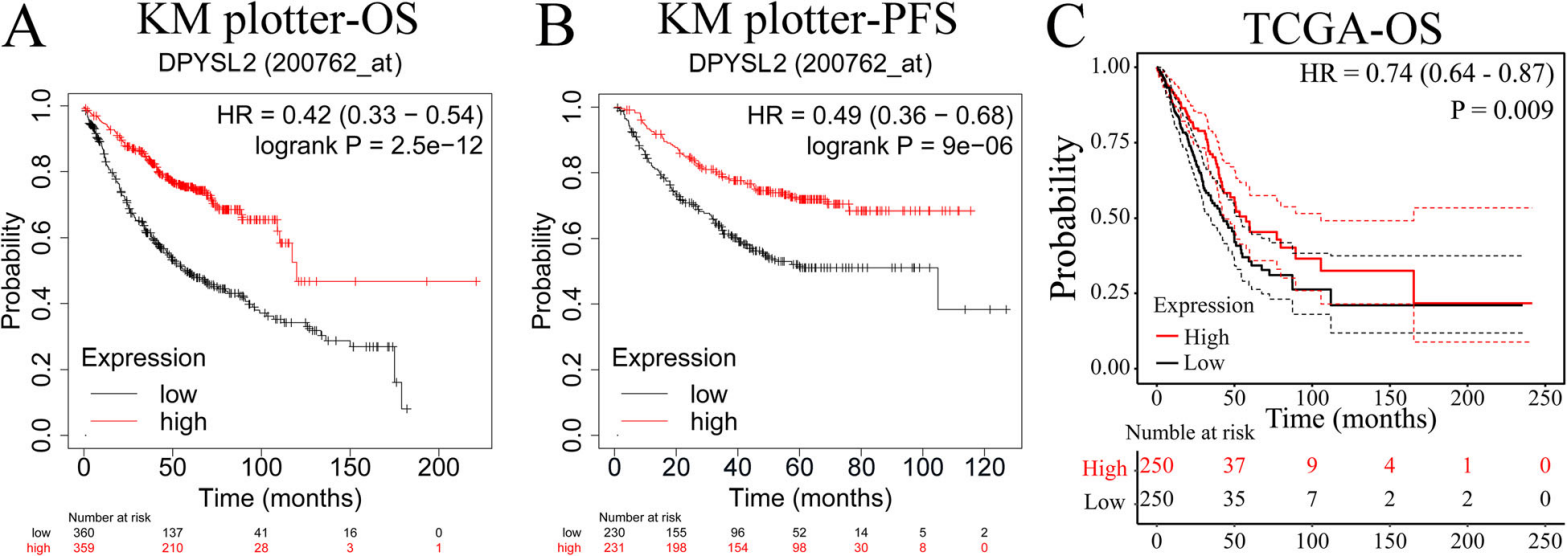
Survival analysis of DPYSL2 in LUAD in the Kaplan–Meier plotter databases and TCGA-LUAD cohort. Kaplan–Meier survival curves of DPYSL2 in overall survival (**A**) and progression-free survival (**B**), overall survival analysis of DPYSL2 in TCGA-LUAD cohort analyzed in R (version 4.0.3) (**C**)

Data were analyzed using the Kaplan–Meier Plotter. Patients were classified in relation to the median DPYSL2 expression levels. Notably, low DPYSL2 expression exhibited a significant correlation with poor OS (Fig. 3A; OS; hazard ratio HR = 0.42, 95% CI = 0.33–0.54, log-rank *P =* 2.5e−12) and PFS (Fig. 3B; PFS; HR = 0.49, 95% CI = 0.36–0.68, log-rank *P =* 9e−06) in LUAD patients. Similarly, low DPYSL2 expression was linked with adverse OS in TCGA-LUAD cohort (Fig. 3C; OS; HR = 0.74, 95% CI = 0.64–0.87, log-rank *P =* 0.009).

To further elucidate the relevance of low DPYSL2 expression on survival, we explored the interrelation in the DPYSL2 expression with clinical characteristics of LUAD patients. Low DPYSL2 expression had a correlation with worse PFS and OS in males and females, stage I, stage M0, and in smoking and nonsmoking LUAD patients (Table 1; *P* < 0.05). Furthermore, we found that low expression predicted poor OS in stage 2 and stage N0 patients, and PFS in stage T1 and stage N1 patients (Table 1; *P* < 0.05).

**Table 1:** The association between DPYSL2 expression and prognosis in LUAD based on Kaplan–Meier plotter analysis

	Overall survival (*n* = 719)				Progression-free survival (*n* = 641)		
	*N*	HR (95% CI)	*p* value		*N*	HR (95% CI)	*p* value
Gender							
Female	317	0.29 (0.19–0.45)	**2.3e−09**		235	1.99 (1.25–3.17)	**0.0032**
Male	344	0.49 (0.34–0.68)	**2.6e−05**		226	2.96 (1.86–4.7)	**1.5e−06**
Stage							
1	370	0.34 (0.22–0.52)	**2.1e−07**		283	2.75 (1.65–4.61)	**5.8e−05**
2	136	0.43 (0.26–0.72)	**8e−04**		103	1.38 (0.8–2.4)	0.25
3	24	0.72 (0.25–2.07)	0.54		10	–	–
4	4	–	–		0	–	–
T stage							
1	123	0.67 (0.36–1.24)	0.2		47	7.47 (0.89–62.42)	**0.029**
2	105	0.77 (0.44–1.33)	0.34		93	1.59 (0.84–3.01)	0.15
3	4	–	–		2	–	–
4	0	–	–		0	–	–
N stage							
0	184	1.87 (1.14–3.05)	**0.011**		102	1.49 (0.68–3.25)	0.31
1	44	1.18 (0.54–2.57)	0.68		38	3.16 (1.23–8.13)	**0.012**
2	3	–	–		2	–	–
M stage							
0	231	1.56 (1.04–2.34)	**0.028**		142	2.17 (1.19–3.93)	**0.0093**
1	1	–	–		0	–	–
Smoke							
Ever	246	2.03 (1.25–3.28)	**0.0033**		243	2.29(1.46–3.6)	**0.00021**
Never	143	3.42 (1.35–8.62)	**0.0057**		143	3.1 (1.58–6.07)	**5e−04**

*HR* hazard ratio, *CI* confidence interval. Values in bold indicate *p* < 0.05

R packages (survminer” and “survival”) were used to fit the Cox regressive models. We applied the Cox analyses to establish the prognostic significance of DPYSL2 expression in the TCGA-LUAD dataset. The potential OS-related variables, such as stage, T classification, N classification, M classification, and DPYSL2 were revealed through Univariate Cox analysis (Table 2; *P* < 0.05). In the multivariate analysis, T classification (HR = 1.278, 95% CI = 1.016–1.608, *P =* 0.036) and DPYSL2 expression (HR = 0.778, 95% CI = 0.648–0.934, *P =* 0.007) could independently predict reduced OS among patients with LUAD (Table 2).

**Table 2:** Cox analyses (univariate and multivariate) of overall survival in the TCGA-LUAD cohort

Variable	Univariate analysis		Multivariate analysis	
	HR (95% CI)	***p*** value	HR (95% CI)	***p*** value
Age (< 65 vs > 65 years)	1.097 (0.781–1.540)	0.594		
Gender				
Female vs male	1.059 (0.756–1.483)	0.739		
Stage				
I vs II vs III vs IV	1.577 (1.348–1.845)	< 0.001	1.352 (0.902–2.025)	0.144
T classification				
T1 vs T2 vs T3 vs T4	1.579 (1.296–1.923)	< 0.001	1.278 (1.016–1.608)	0.036
N classification				
N0 vs N1 vs N2 vs N3	1.706 (1.405–2.072)	< 0.001	1.208 (0.858–1.701)	0.278
M classification				
M0 vs M1	1.843 (1.038–3.272)	0.037	0.912 (0.330–2.517)	0.858
DPYSL2 expression				
High vs low	0.760 (0.637–0.906)	0.002	0.778 (0.648–0.934)	0.007

*HR* hazard ratio, *CI* confidence interval

### DPYSL2 level is linked to immune infiltration in LUAD

**Fig. 4:**
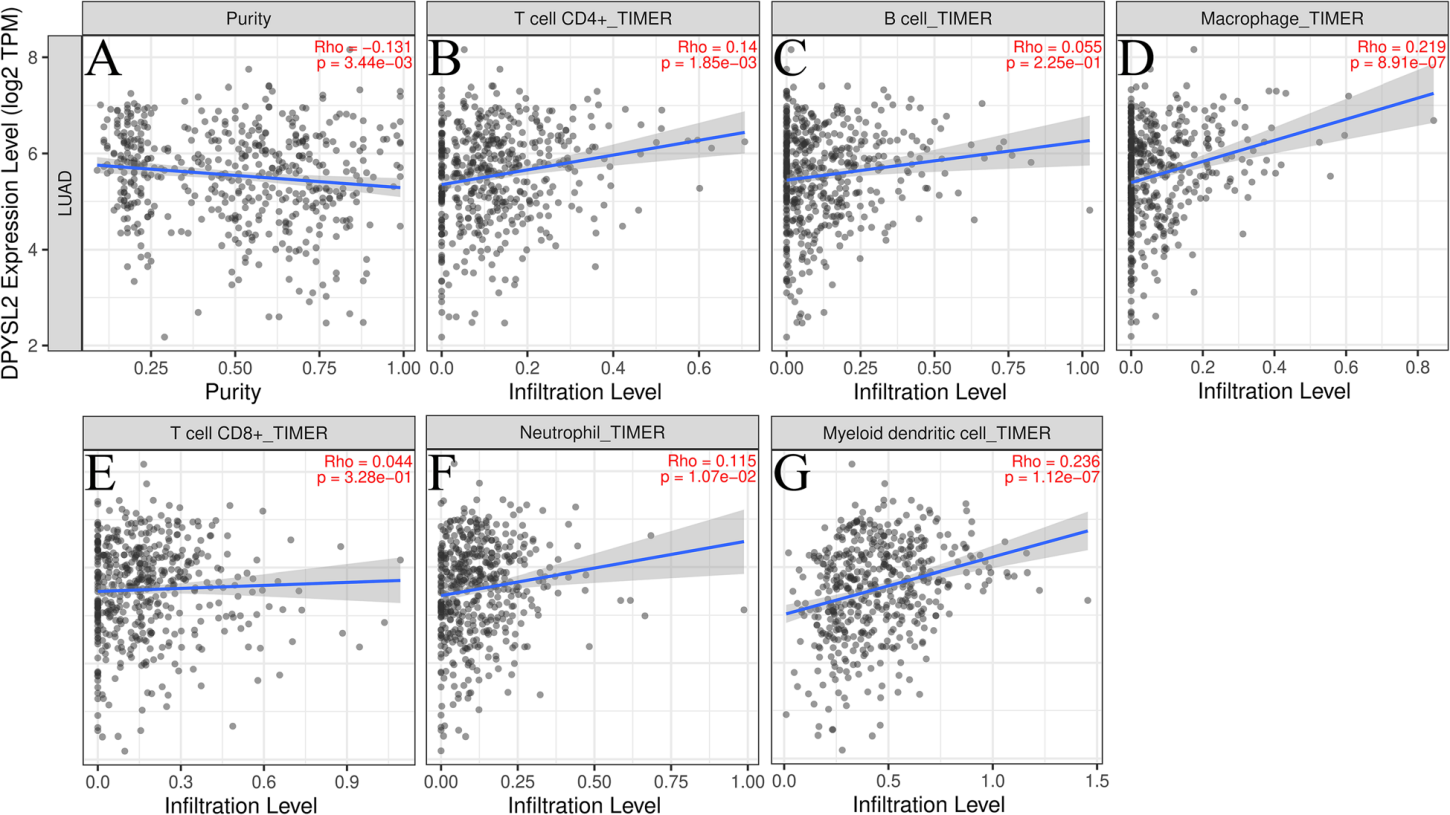
The association between DPYSL2 expression and immune infiltration level in LUAD. Expression of DPYSL2 is inversely linked to tumor purity (**A**) and positively related to CD4+ T cells, macrophages, dendritic cells, and neutrophils in LUAD (**B**–**G**)

Immune infiltration of LUAD potentially influences patient’s survival. Herein, we analyzed associations among DPYSL2 expression, tumor purity, and immune infiltration in the online tool TIMER. The association of DPYSL2 expression with LUAD tumor purity was revealed (Fig. 4A). Also, the expression of DPYSL2 had a beneficial impact on infiltrating levels of CD4+ T cells (*r* = 0.14, *P* = 1.85e−03), macrophages (*r* = 0.219, *P* = 8.91e−07), dendritic cells (*r* = 0.236, *P* = 1.12e−07), and neutrophils (*r* = 0.115, *P* = 1.07e−2) in LUAD, but had no relationship with CD8+ T cells (*r* = 0.044, *P* = 3.28e−01), and B cells (*r* = 0.055, *P* = 2.25e−01) (Fig. 4B–G).

To further explore how DPYSL2 expression is related to infiltrated immune cells, we utilized numerous markers for the characterization of immune cells. Moreover, we included T cells with diverse functions, including Th1, Th2, Tfh, Th17, Treg, and exhausted T cells. A larger proportion of T cells (CD4+ T, Th2, Tfh, etc.), macrophage cells, neutrophils, and dendritic cells were positively associated with DPYSL2 after adjusting for purity (Table 3; *P* < 0.05). We observed that 4 of the 9 irrelevant markers were from CD8+ T cells and B cells, which also supported our previous results. The findings demonstrated that DPYSL2 is crucial in immune infiltration in LUAD.

**Table 3:** Correlation analysis of DPYSL2 and related genes and immune cells markers in TIMER

Description	Gene markers	Non-adjusted		Tumor purity adjusted	
		Cor	***P*** value	Cor	***P*** value
**CD4**^**+**^**T cell**	CD4	0.425	***	0.412	***
**Th1**	STAT4	0.154	**	0.106	1.9e−02
	TNF	0.138	*	0.083	6.63e−02
	TBX21	0.105	1.71e−02	0.06	1.8e-01
**Th2**	STAT5A	0.334	***	0.305	***
	STAT6	0.333	***	0.353	***
	GATA3	0.126	*	0.071	1.17e−01
**Tfh**	CCL13	0.307	***	0.271	***
	BCL6	0.272	***	0.268	***
	CCR5	0.218	***	0.17	**
**Th17**	STAT3	0.35	***	0.349	***
	RORC	0.163	**	0.194	***
	IL17A	− 0.094	3.32e−02	− 0.122	*
**Treg**	STAT5B	0.374	***	0.356	***
	CCR8	0.219	***	0.175	***
	FOXP3	0.133	*	0.08	7.73e−02
**T cell exhaustion**	HAVCR2	0.225	***	0.187	***
	GZMB	− 0.262	***	− 0.333	***
	CTLA4	0.07	1.15e−01	0.003	9.43e−01
	PDCD1	−0.044	3.19e−01	− 0.113	1.24e−02
**CD8**^**+**^**T cell**	CD8A	−0.02	6.52e−01	− 0.08	7.73e−02
	CD8B	−0.116	*	− 0.17	**
**M1 Macrophage**	IRF5	0.15	**	0.118	*
	NOS2	0.13	*	0.116	1e−02
	PTGS2	0.106	1.65e−02	0.115	1.09e−02
**M2 Macrophage**	MS4A4A	0.306	***	0.28	***
	CD163	0.291	***	0.267	***
	VSIG4	0.241	***	0.215	***
**Neutrophil**	CEACAM8	0.428	***	0.428	***
	ITGAM	0.383	***	0.362	***
	CCR7	0.266	***	0.229	***
**Dendritic cell**	CD1C	0.522	***	0.499	***
	HLA-DPB1	0.405	***	0.388	***
	HLA-DPA1	0.401	***	0.382	***
	ITGAX	0.29	***	0.268	***
**TAM**	CD68	0.234	***	0.212	***
	IL10	0.164	**	0.114	1.13e−02
	CCL2	0.162	**	0.113	1.17e−02
**B cell**	CD27	0.077	8.08e−02	0.027	5.48e−01
	CD19	0.057	1.94e−01	0.009	8.38e−01
	CD79A	0.052	2.34e−01	0.011	8.11e−01

*Cor* correlation coefficient; *P < 0.05, **P < 0.01, ***P < 0.001

### DPYSL2 mediates immune activation and response in LUAD

**Fig. 5:**
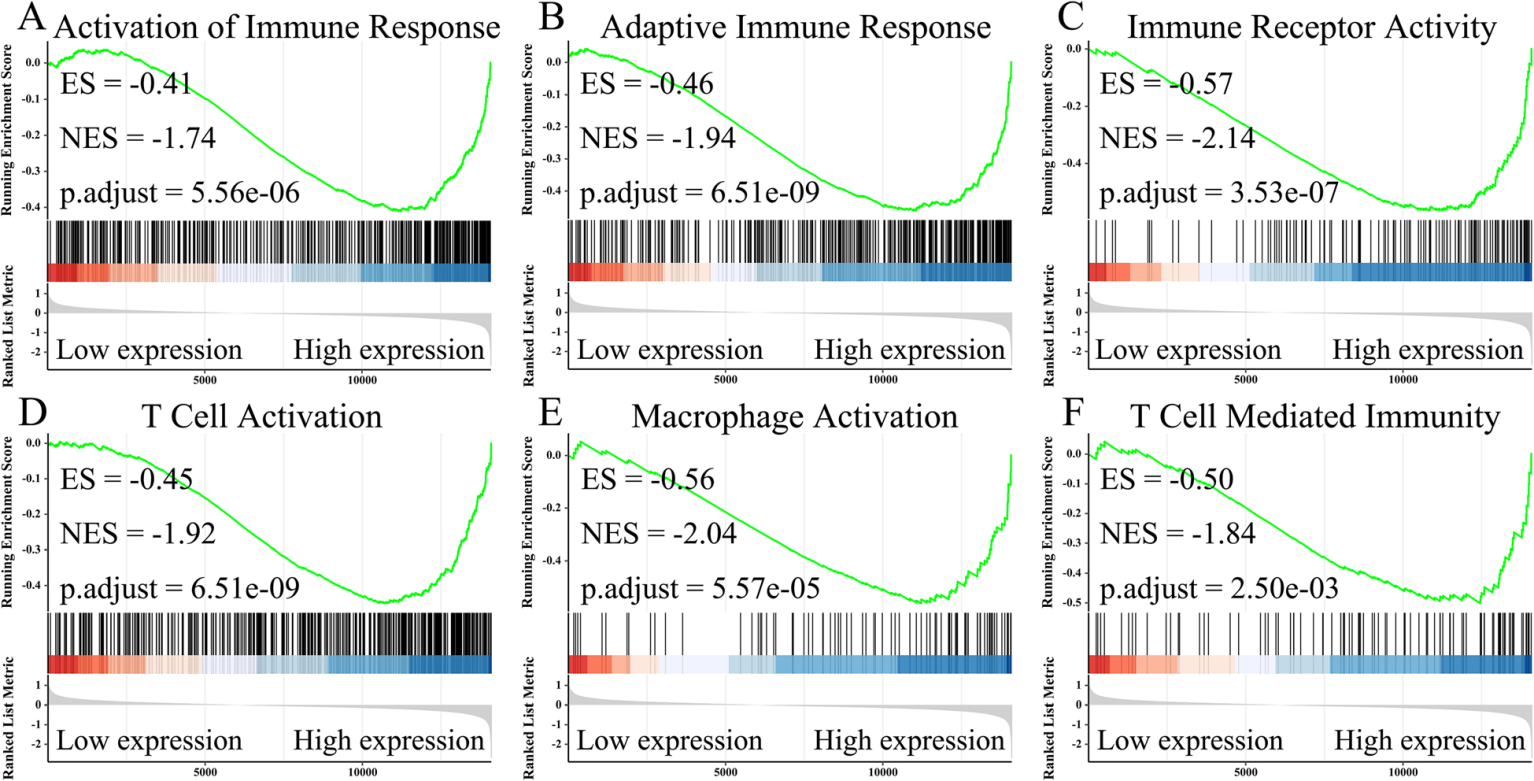
Immune-related signal pathways enriched in the DPYSL2 high expression group of LUAD. Immune-related signal pathways enriched in LUAD patients with high DPYSL2 phenotype were activation of immune response (**A**), adaptive immune response (**B**), immune receptor activity (**C**), T cell activation (**D**), macrophage activation (**E**), and T cell-mediated immunity (**F**)

Based on the previous results, we hypothesized that high expression of DPYSL2 exerts effects in the prevention of the onset and progression of LUAD. Our study investigated the possible mechanism of DPYSL2 in LUAD. High and low expression groups were obtained from TCGA-LUAD cohort based on DPYSL2 levels. For the TCGA dataset analysis, we applied GSEA. Interestingly, we found that many gene-sets linked to immune activation and response, such as activation of the immune response (Fig. 5A; *adjusted P* = 5.56e−06), adaptive immune response (Fig. 5B; *adjusted P* = 6.51e−09), immune receptor activity (Fig. 5C; *adjusted P* = 3.53e−07), T cell activation (Fig. 5D; *adjusted P* = 6.51e−09), macrophage activation (Fig. 5E; *adjusted P* = 5.57e−09), and T cell-mediated immunity (Fig. 5F; *adjusted P* = 2.50e−03), were enriched in the DPYSL2 high expression group. Hence, we hypothesized that DPYSL2 inhibits the progression of LUAD through immune activation and response during the tumor process.

## Discussion

Dihydropyrimidinase like 2 (DPYSL2) participates in the formation of the cytoskeleton and has a close association with the development of neurons [18, 32, 33]. Studies have shown that DPYSL2 affects tumor cell migration via its effect on microtubules; however, it impedes stemness and metastasis of breast cancer cells when combined with RECK [20, 21, 34]. These implicate DPYSL2 in tumor metastasis and drug resistance is complex. However, there is still a lack of extensive and in-depth research on the role of DPYSL2 in tumors. In this study, we uncovered that a low DPYSL2 level is linked to a poor prognosis of LUAD patients. We also identified that the level of DPYSL2 expression in the LUAD microenvironment is associated with immune cells and their specific immune markers. Different degrees of immune infiltration led to different immunotherapy curative effects in LUAD patients. Taken together, these findings indicate that DPYSL2 will likely become a new tumor marker and immunotherapy target in LUAD.

Our investigations of multiple databases such as Oncomine, TIMER, and TCGA, revealed that, in comparison to the adjacent normal tissues, DPYSL2 expression was notably lower in LUAD tissues. Additionally, ROC curves indicated that the AUC value of DPYSL2 was close to 1. These results revealed that the expression of DPYSL2 has high sensitivity and specificity, and high diagnostic value in distinguishing LUAD patients from healthy individuals. Clinically, OS and PFS are frequently used to assess the prognosis of patients ailing from cancer. For prognosis, low DPYSL2 levels were linked to the poor OS and PFS. Furthermore, the DPYSL2 level was found to affect the OS in the TCGA-LUAD cohort. Consequently, our data supported the prognostic value of DPYSL2 as a biomarker in LUAD.

The recent addition of immunotherapy has resulted in tremendous improvements in cancer treatment models [35, 36]. Moreover, the function driven by the immune system in tumorigenesis and progression is becoming well understood [37–39]. Analyzing the types and numbers of immune cells present in tumors, will aid in selecting immunotherapy drugs and predicting their effectiveness, and is expected to reveal new therapeutic targets [40–42].

The present findings demonstrate that a correlation exists between DPYSL2 expression and neutrophils, macrophages, CD4+ T cells, and dendritic cells in the tumor microenvironment of LUAD. Moreover, DPYSL2 is associated with LUAD immune cell molecular markers, implying that DPSYL2 has a role in tumor immune regulation in LUAD. Particularly, several CD4+ T helper cell markers (CD4, STAT4, STAT5A, STAT6, CCL13, BCL6, CCR5, STAT3, RORC, STAT5B, CCR8) exhibit a significant positive correlation with the DPYSL2 expression. Scholars have reported that CD4+ T cells are critically vital in mediating tumor cell killing via multiple pathways [43–45]. Therefore, these correlations may highlight a mechanism by which DPYSL2 potentially tune the functions of T cells in LUAD. In addition, dendritic cell markers (CD1C, HLA-DPA1, HLA-DPB1, ITGAX) were also significantly related to the expression of DPYSL2. According to studies, dendritic cells present tumor-related antigens and provide immune regulatory signals to activate T cells in TME, thereby enhancing tumor immunity [46]. Moreover, macrophage markers (IRF5, NOS2, PTGS2, MS4A4A, CD163, VSIG4, CD68, IL10, CCL2) exhibited a positive correlation with DPYSL2 expression. M1 macrophages stimulate the immune response by secreting pro-inflammatory cytokines, whereas M2 macrophages may play the opposite role by secreting anti-inflammatory cytokines and promoting angiogenesis [47, 48]. However, when stimulated simultaneously, they can inhibit tumor progression by collaborating with T cells [49].

We used GSEA to analyze TCGA data to have an in-depth understanding of the function of DPYSL2 in LUAD, especially the function related to immune infiltration. We found that DPYSL2 is significantly linked with activation of an immune response, immune receptor activity, macrophage activation, T cell activation, and T cell-mediated immunity. Collectively, the present findings imply that DPYSL2 can recruit and regulate immune infiltrating cells in LUAD.

This study has significantly enhanced our understanding of the relationship between DPYSL2 and LUAD; however, it does not identify the exact mechanism of DPYSL2 and immune infiltrating cells, thus calling for further research.

## Conclusion

In conclusion, an association exists between DPYSL2 expression and poor prognosis in LUAD patients, and the underlying mechanism may be related to immune response. By recruiting and regulating a variety of immune infiltrating cells, DPYSL2 may improve the prognosis of LUAD patients. These findings may lead to the design of effective LUAD diagnostic biomarkers and immunotherapy.

## Data Availability

The datasets used during the current study are available from the corresponding author on reasonable request.
